# F-actin Regulates Osteoblastic Differentiation of Mesenchymal Stem Cells on TiO_2_ Nanotubes Through MKL1 and YAP/TAZ

**DOI:** 10.1186/s11671-020-03415-9

**Published:** 2020-09-23

**Authors:** Zhicheng Tong, Yanchang Liu, Runzhi Xia, Yongyun Chang, Yi Hu, Pengcheng Liu, Zanjing Zhai, Jingwei Zhang, Huiwu Li

**Affiliations:** 1 Shanghai Key Laboratory of Orthopaedic Implants, Department of Orthopaedic Surgery, Shanghai Ninth People’s Hospital, Shanghai Jiao Tong University School of Medicine, Shanghai, 200011 People’s Republic of China; 2 Department of Orthopedics, The Second Hospital of Anhui Medical University, Hefei, 230601 Anhui People’s Republic of China

**Keywords:** TiO_2_ nanotubes, MSCs, Osteogenic differentiation, F-actin, MKL1, YAP/TAZ

## Abstract

Titanium and titanium alloys are widely used in orthopedic implants. Modifying the nanotopography provides a new strategy to improve osseointegration of titanium substrates. Filamentous actin (F-actin) polymerization, as a mechanical loading structure, is generally considered to be involved in cell migration, endocytosis, cell division, and cell shape maintenance. Whether F-actin is involved and how it functions in nanotube-induced osteogenic differentiation of mesenchymal stem cells (MSCs) remain to be elucidated. In this study, we fabricated TiO_2_ nanotubes on the surface of a titanium substrate by anodic oxidation and characterized their features by scanning electron microscopy (SEM), X-ray energy dispersive analysis (EDS), and atomic force microscopy (AFM). Alkaline phosphatase (ALP) staining, Western blotting, qRT-PCR, and immunofluorescence staining were performed to explore the osteogenic potential, the level of F-actin, and the expression of MKL1 and YAP/TAZ. Our results showed that the inner diameter and roughness of TiO_2_ nanotubes increased with the increase of the anodic oxidation voltage from 30 to 70 V, while their height was 2 μm consistently. Further, the larger the tube diameter, the stronger the ability of TiO_2_ nanotubes to promote osteogenic differentiation of MSCs. Inhibiting F-actin polymerization by Cyto D inhibited osteogenic differentiation of MSCs as well as the expression of proteins contained in focal adhesion complexes such as vinculin (VCL) and focal adhesion kinase (FAK). In contrast, after Jasp treatment, polymerization of F-actin enhanced the expression of RhoA and transcription factors YAP/TAZ. Based on these data, we concluded that TiO_2_ nanotubes facilitated the osteogenic differentiation of MSCs, and this ability was enhanced with the increasing diameter of the nanotubes within a certain range (30–70 V). F-actin mediated this process through MKL1 and YAP/TAZ.

## Introduction

Titanium and titanium alloys, due to their excellent biocompatibility, corrosion resistance, and mechanical properties, are widely used in clinical applications such as total joint replacements and dental implants [1–3]. However, there are still many challenges remaining to be solved, including aseptic loosening and infection [4, 5]. In recent years, a number of studies aimed at enhancing osseointegration and antibacterial properties have been carried out. For example, the MoS_2_/PDA-RGD coating on titanium implants can not only promote the integration of a titanium implant with the host bone but also inhibit bacterial growth with a high efficiency [6]. In addition, the surface topography has attracted more and more attention, and topographical modification differs from chemical modifications by only changing the micro- and nano-scale structure. The stimulation of chemical signals on cells is unstable and cytotoxic. In comparison, safe and controllable physical signals can avoid some side effects caused by chemical molecules. Therefore, topographical modification of the surface of implants and the regulation of osseointegration through topographical structure can provide a new way to solve the clinical problem of poor osseointegration after implantation of prostheses.

In the field of bone tissue engineering and bone regeneration, cell-morphology interaction is considered to be a promising management strategy for precise control of seed cell function and differentiation. At the same time, the bone itself has an elegant hierarchy within the nanometer and micron range [7]. Therefore, surface morphology can provide a similar niche, which can mimic natural bone structure and promote osteogenic differentiation of mesenchymal stem cells on the surface of the host bone and implant. Surface morphologies can consist of many different structures, including nanotubes, nanowires, nanopores, and so on. In particular, nanotube arrays have attracted extensive interest in many fields in recent years due to their unique surface characteristics, such as high surface-to-volume ratio, biological plasticity, and high adsorption capacity. For example, a new study shows that boron nitride nanotubes (BNNT) constitute a gas-sensitive material that can be used as a gas sensor to monitor the operation of transformers by detecting the composition and content of dissolved gases in oil [8]. In biomedicine, surface topography is also able to direct cellular behaviors, including cell migration, adhesion, proliferation, and differentiation. The latest studies reveal that nanoscale topography can direct mesenchymal stem cells (MSCs) to differentiate into osteoblasts so as to reinforce early osseointegration [9–12]. It is even reported that combined micro- and nano-scale surface modification can cause MSCs to differentiate into contractile smooth muscle cells [13]. However, the molecular mechanisms of how surface topography directs cell fate have yet to be elucidated, which is important for material safety evaluation and material design.

Filamentous (F)-actin, also called microfilament, is one of the three major components of the cytoskeleton in eukaryotic cells. It is composed of polymers of globular (G)-actin, modified by numerous other proteins. F-actin has structural polarity due to the fact that all the microfilament’s subunits point toward the same end. The barbed end is directed at a different adjacent monomer, while the pointed end possesses an actin subunit with the ATP binding site exposed. That is, ATP is involved in the process of transformation between G-actin and F-actin. This process is in a dynamic equilibrium, with polymerization and depolymerization occurring simultaneously, also known as treadmilling, often seen in lamellipodia and filopodia [14]. Therefore, it is obvious that actin dynamics play an important role in such cellular functions as cell migration, cell division, and maintenance of cell shape. However, F-actin not only acts as a physical structure supporting mechanical loading but also participates in other biological behaviors such as signal transduction and gene expression. Accumulating evidence demonstrates that F-actin can convert physical signals into chemical signals by interacting with other proteins [15–18]. For instance, biomechanical and geometric reconstruction promote tumor cell apoptosis by preventing the superposition of actin monomer polymerization to F-actin [15]. Ultrasound pulses enhance osteogenesis of human mesenchymal stem cells by inhibiting depolymerization of F-actin [16]. Our previous study also showed that mechanical strain increases the stability of F-actin [17]. Because the hollow structure of nanotubes provides fewer adhesion sites for cells, rearrangement of the cytoskeleton is inevitable in order to maintain the biomechanical balance. Consequently, we definitely have reason to believe that F-actin is likely to mediate nanotopography-induced cell differentiation.

In this study, we fabricated TiO_2_ nanotubes, modified their topography by anodic oxidation, and explored their ability to promote osteogenic differentiation of MSCs. Next, we investigated whether F-actin plays a critical role in mechanotransduction. Cytochalasin D (Cyto D), which competitively binds to the barbed end of F-actin to prevent G-actin from incorporating into the filament, was used to inhibit F-actin polymerization, and jasplakinolide (Jasp) was used to enhance stabilization of the actin assembly. Furthermore, we also wanted to elucidate how F-actin functions in changing physical cues into biochemical signals. Based on the results of our previous study, we hypothesized that the MAPK pathway may be involved in this process [17]. Transcription factors such as Yes-associated protein (YAP)/transcriptional coactivator with PDZ-binding motif (TAZ) and MKL1, which are considered to be mechanosensors and mechanotransducers, were also the subjects of our study to screen how F-actin influences stem cell fate, because some studies in other areas implied that they were related to F-actin [19–21]. In general, we hope to clarify the role of F-actin in the process of stem cell differentiation induced by nanotubes, so as to guide the material design and biosafety assessment of implants modified by nanotubes.

## Materials and Methods

### Fabrication of TiO_2_ Nanotubes

Pure titanium slices (99.9% purity, 2 mm thickness; Shengshida, Hebei, China), used as the substrate, were polished with silicon carbide sandpaper of No. 400 and 1500 grits. Samples were then washed sequentially with acetone, anhydrous alcohol, and deionized water in an ultrasonic cleaning machine, and finally dried at room temperature for 3 h. To fabricate the nanotopography, the pretreated samples were fixed as the anode, while using a platinum piece as the counter cathode in an electrolyte aqueous solution of 0.15 M NH_4_F and 90% glycol for 1 h. The anodization voltage was a constant voltage of 30, 40, 50, 60, or 70 V. After anodic oxidation, every sample was rinsed with deionized water for 30 min and washed with anhydrous alcohol in an ultrasonic cleaning machine for 15 min. Finally, all samples were sterilized in an autoclave at 120 °C for 1 h then moistened with culture medium before use.

**Fig. 1:**
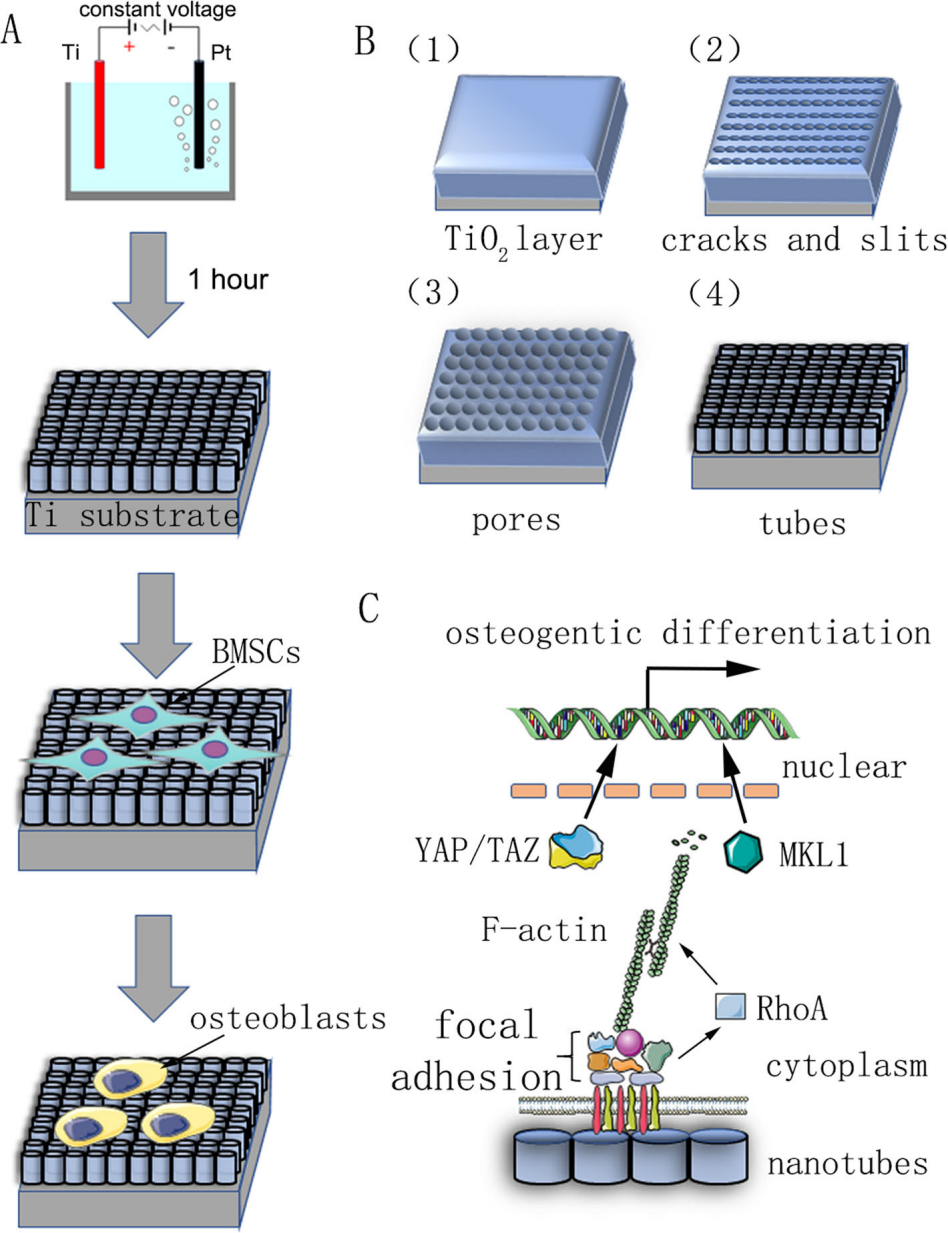
A summary chart of our study. **a** Flow chart showing anodic oxidation and induction of cell differentiation. **b** The pretreated titanium substrate was fixed as the anode in an electrolyte aqueous solution with 0.15 M NH_4_F and 90% glycol at constant voltage for 1 h. Self-assembled nanotubes were predicted to be formed uniformly. Its reaction mechanism is described in materials and methods. **c** Schematic diagram of the mechanism of osteogenic differentiation of stem cells induced by nanotubes

The reaction mechanism of nanotube fabrication is not clear, and the current mainstream theory is field-enhanced dissolution theory. The formation of nanotube arrays is the result of dynamic equilibrium under the action of field oxidation, field dissolution, and chemical dissolution (Fig. 1b). The anodization process can be described as follows: in the first step, an oxide barrier layer is formed on the electrolyte–metal interface:

1 $$ {\mathrm{Ti}}^{4+}+2{\mathrm{H}}_2\mathrm{O}\to {\mathrm{Ti}\mathrm{O}}_2+4{\mathrm{H}}^{+} $$

Then cracks and narrow slits appear on the surface due to field-enhanced dissolution of the oxide layer. Diffusion of F^−^ ions into these cracks and slits enhances the dissolution rate. Cracks enlarge and become connected with neighboring cracks. Finally, the formation rate and dissolution rate of the titanium oxide layer reach a dynamic balance, and the nanotubes no longer grow:

2 $$ {\mathrm{TiO}}_2+6{\mathrm{F}}^{-}+4{\mathrm{H}}^{+}\to {\left[{\mathrm{TiF}}_6\right]}^{2-}+2{\mathrm{H}}_2\mathrm{O} $$

### Surface Characterization

Samples fabricated with different voltages (30, 40, 50, 60, or 70 V) were rinsed with ethanol and deionized water for 15 min, then dried at room temperature. Scanning electron microscopy (SEM450, FEI Nova Nano SEM; Thermo Fisher Scientific, Waltham, MA, USA) was used to characterize surface structure, and to measure the inner diameter and height of the nanotubes after coating samples with a thin layer of gold. Meanwhile, X-ray energy dispersive analysis (EDS) was performed to analyze the elemental composition of nanotubes. Atomic force microscopy (AFM, NanoManVS, Bruker Nano Surfaces, Bruker MicroCT, Kontich, Belgium) was used to investigate the surface morphology and surface roughness of the samples. Three different areas were selected from each sample and the measurements were repeated three times.

### Cell Culture

Four-week-old male Sprague-Dawley (SD) rats were purchased from the experimental animal center of Shanghai Ninth People’s Hospital (Shanghai, China). Rat bone marrow mesenchymal stem cells (BMSCs) were aseptically isolated from femurs and tibias. BMSCs were purified and further expanded in α-minimal essential medium (α-MEM; Hyclone, Logan, UT, USA) containing 10% (v/v) fetal bovine serum (FBS) (Gibco/Life Technologies, Carlsbad, CA, USA), 100 mg/mL streptomycin (Gibco), and 100 U/mL penicillin (Gibco), and incubated at 37 °C in a humidified atmosphere consisting of 95% air and 5% CO_2_. The culture medium was renewed every 2 days, and cells were trypsinized and subcultured at 80% confluence. All cells used in this study were between passages 3 and 5. Osteogenic induction medium was composed of growth medium supplemented with 100 nM dexamethasone, 10 mM β-glycerophosphate, and 50 mM ascorbic acid (Sigma–Aldrich, St Louis, MO, USA).

### Cell Proliferation Assay

The TiO_2_ nanotube-modified titanium slices were cut into circular shapes and placed into wells of a 24-well cell culture plate. BMSCs between passages 3–5 were cultured on TiO_2_ nanotubes at a density of 3 × 10^4^ cells/disc in either growth medium or osteogenic medium. After 2 days of cell culture, cytochalasin D (Cyto D, Sigma–Aldrich) and jasplakinolide (Jasp, Sigma–Aldrich), used to disrupt F-actin polymerization, were added to the medium every day for 3 days. The final concentration and working time of Cyto D and Jasp were 5 μM, 1 h and 2 μM, 3 h respectively. The culture medium was renewed after incubation with the reagents. Cell viability and proliferation were assessed using the Cell Counting Kit-8 (CCK8) assay (Dojindo, Kumamoto, Japan) 12 h after Cyto D or Jasp treatment. The cells were incubated with 10% (v/v) CCK8 solution for 2 h in a cell incubator at 37 °C under 5% CO_2_. Then we transferred 100 μL of the reaction mixture into wells of a 96-well plate and absorbance (OD) of the formazan dye product in the culture was measured at 450 nm using a Multiscan UV–visible spectrophotometer (Safire2; TECAN, Mannedorf, Switzerland). In addition, we also counted approximate cell numbers using an automated cell counter (AMQAX1000, Life Technologies). Before cell counting, BMSCs were enzymatically detached from TiO_2_ nanotubes and stained with Trypan blue (Sigma–Aldrich).

### Alkaline Phosphatase Staining and ALP Activity Analysis

BMSCs were seeded onto five different TiO_2_ nanotube-modified titanium slices (30, 40, 50, 60, or 70 V) at a density of 3 × 10^4^ per well and cultured in osteogenic medium. Reagents were added as described above. After 7 days of incubation, BMSCs cultured on TiO_2_ nanotubes were washed three times with PBS, fixed with 4% paraformaldehyde, and incubated in alkaline phosphatase (ALP) working solution of an ALP kit according to the manufacturer’s instructions (Hongqiao, Shanghai, China). The results were observed under a stereo microscope after washing with PBS.

For ALP activity analysis, cells were first lysed with RIPA buffer without protease and phosphatase inhibitors, and then the centrifuged lysates were assayed using an ALP Assay Kit (Beyotime Institute of Biotechnology, Jiangsu, China) following the protocol provided. The activity was finally normalized to the protein concentration of the corresponding lysate.

### Immunocytochemistry

After 3 days of Cyto D and Jasp treatment, BMSCs were fixed with 4% paraformaldehyde for 20 min at room temperature and then washed three times with PBS. The cells were permeabilized with 0.3% Triton-X 100 for 30 min, washed three times with PBS, and stained with Rhodamine-conjugated phalloidin for 1 h at room temperature in the dark. Then cells were rinsed with PBS, and counterstained with DAPI (Beyotime Institute of Biotechnology) for 10 min at room temperature. Following three additional washes with PBS, samples were fixed on a glass slide and observed by confocal microscopy.

### Western Blotting

To evaluate protein expression, BMSCs cultured on TiO_2_ nanotubes were harvested with trypsin (Gibco). The cells were washed three times with PBS and lysed with RIPA buffer supplemented with a protease and phosphatase inhibitor cocktail for 30 min on ice. The lysate was collected by centrifugation at 12,000×*g* for 15 min at 4 °C. The concentration of total proteins in the supernatant was measured using a bicinchoninic acid (BCA) protein assay kit (Beyotime) according to the manufacturer’s instructions. Loading buffer was added to protein samples as above, which were then boiled at 95 °C for 15 min. For Western blotting analysis, 10 μL of the protein preparation was loaded onto a 12.5% SDS-PAGE gel (EpiZyme Inc., Cambridge, MA, USA) and subjected to electrophoresis at 120 V for 1 h, then electro-transferred onto a polyvinylidenedifluoride (PVDF) membrane at 250 mA for 2 h. The membranes were then blocked with 5–10% non-fat dried milk in TBST for 1 h on a shaker at room temperature and incubated with primary antibody diluted in dilution buffer (Beyotime) at 4 °C overnight. Next, fluorescent-conjugated secondary antibody diluted in dilution buffer was added to the membranes after washing three times with TBST for 5 min, and the membranes were then incubated at room temperature for 1 h in the dark. The protein bands were detected by a two-color infrared fluorescence imaging system (Odyssey, LiCor Biosciences, Lincoln, NE, USA). In particular, if the bands of internal reference protein were unified, the membrane was stripped and reprobed with another primary antibody, followed by the same process. We used GAPDH antibody as our internal reference protein to normalize protein expression, and the other primary antibodies used in this study were anti-vinculin (1:1000 dilution, Abcam, Cambridge, MA, USA), anti-FAK (1:1000 dilution, Cell Signaling Technology, Danvers, MA, USA), anti-Runx2 (1:1000 dilution, Cell Signaling Technology), anti-RhoA (1:1000 dilution, Cell Signaling Technology), anti-F-actin (1:500 dilution, Abcam), anti-Osx (1:500 dilution, Abcam), and anti-pYAP (1:1000 dilution, Cell Signaling Technology). Secondary antibodies were goat anti-mouse IgG H&L (IRDye® 680RD, 1:5000 dilution, Abcam) and goat anti-rabbit IgG H&L (IRDye® 680RD, 1:5000 dilution, Abcam).

### Quantitative Real-Time PCR

Quantitative real-time PCR was carried out on day 7 to evaluate the gene expression of runt-related transcription factor 2 (Runx2), Osterix (Osx), Alp, osteocalcin (OCN), RhoA, YAP, TAZ, vinculin (VCL), focal adhesion kinase (FAK), and megakaryoblastic leukemia 1 (MKL1) in cells grown in osteogenic medium on TiO_2_ nanotubes. Total RNA was extracted from the cells using a Total RNA Kit (R6812-01HP, Omega Bio-Tek Inc., Norcross, GA, USA). The concentration and purity of RNA samples were determined by optical density at a wavelength of 260 and only samples presenting both A260/280 ratios and A260/230 ratios higher than 1.8 were analyzed. RNA samples were reverse-transcribed into cDNA using a qScript cDNA Synthesis kit (Takara, Shiga, Japan) according to the manufacturer’s instructions. Quantitative real-time PCR was performed with SYBR® Premix Ex Taq™ (Takara) using a QuantStudio 6 Flex real-time PCR system (Life Technologies). GAPDH, a house-keeping gene, was used as an internal reference. Data were analyzed using the comparison Ct (2^-ΔΔCt^) method and expressed as fold changes compared to the control. The sequences of the primers used are listed in Table 1.

**Table 1:** Primers used in the qRT-PCR assay

Gene	Forward (5′-3′)	Reverse (5′-3′)
GAPDH	GGCAAGTTCAACGGCACAG	CGCCAGTAGACTCCACGACAT
Runx2	CTTCGTCAGCGTCCTATC	CTTCCATCAGCGTCAACA
Osx	GTTCACCTGTCTGCTCTG	GGCTGATTGGCTTCTTCT
Alp	GTGGTATTGTAGGTGCTGTGGTC	CGGTGTCGTAGCCTTCTGG
OCN	GTAAGGTGGTGAATAGACTCC	AACGGTGGTGCCATAGAT
RhoA	ACTGGTGATTGTTGGTGAT	GAACTGGTCCTTGCTGAA
YAP	CATAAGAACAAGACCACATCCT	AATCGCAGCCTCTCCTTC
TAZ	CAAGGAAGTGCTGTATGAG	GTGGTTAGAGACGGTGATA
VCL	CCAAGTGTGACCGTGTAG	GGAGTTGTAGTATCGCTGAA
FAK	GCAGTCCTTCATCATCAGA	CTCCAATACAGCGTCCAA
MKL1	GGTATGAAGAGACTGTGACT	TTCTGCTGGAGGTGACTT

### Statistical Analysis

All data are representative of at least three independent experiments using triplicate samples unless otherwise indicated. Data are expressed as the mean ± standard deviation (SD). Differences between groups were evaluated by one-way analysis of variance followed by the Student-Newman-Keuls post-hoc test or Student’s *t* test. *P* values < 0.05 were considered statistically significant.

## Results

### Surface Characterization

**Fig. 2:**
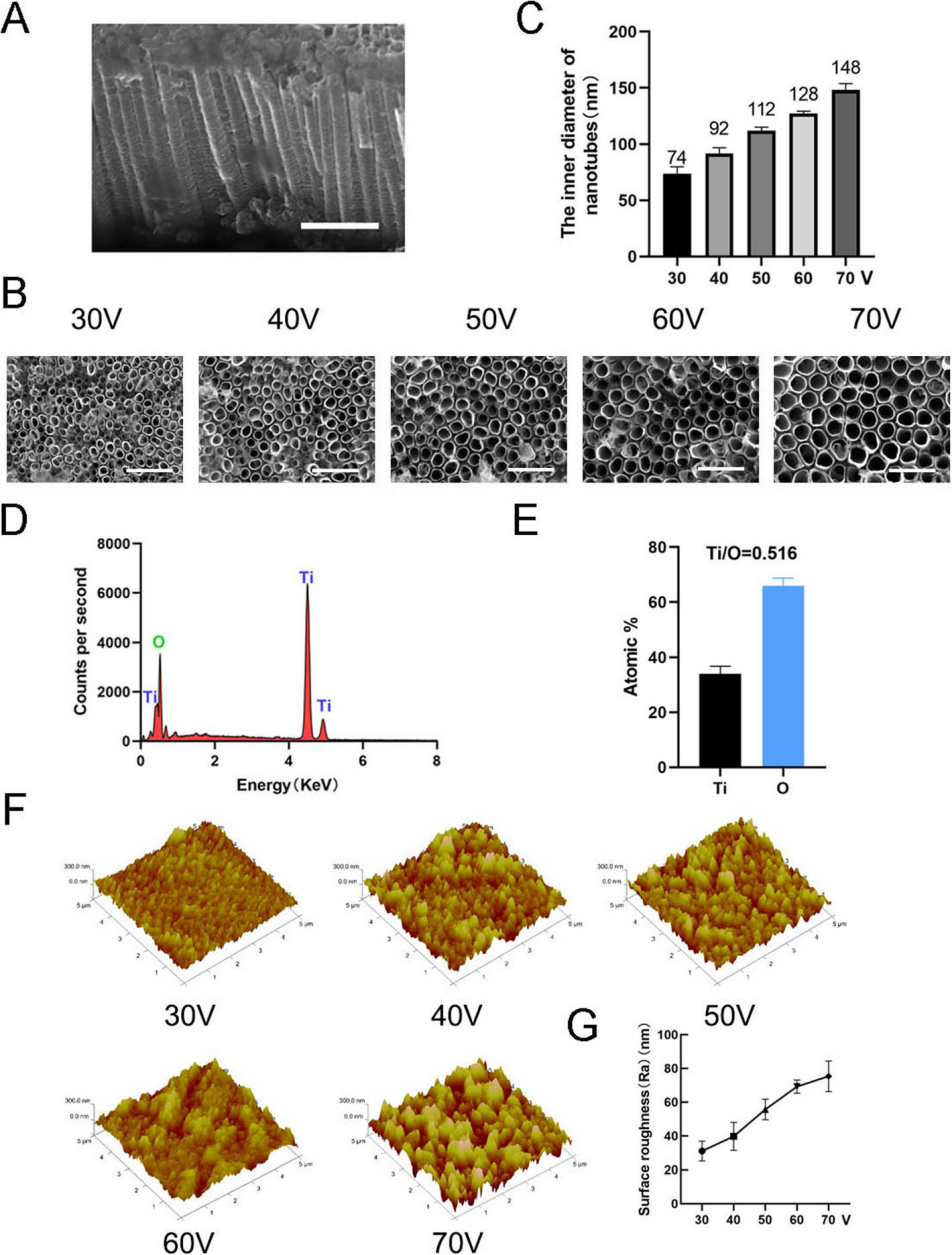
Surface characterization of nanotubes. **a** Side view of nanotubes. Scale bars: 1 μm. **b** Top view of nanotubes. Scale bars: 500 nm. **c** The inner diameter of the nanotubes at five different constant voltages (30, 40, 50, 60, 70 V). **d** The EDS chemical element composition of nanotube structures. **e** The element ratio of Ti and O. **f** Atomic force microscopy (AFM) images of nanotube structures. **g** The average surface roughness (Ra) of the nanotubes

To fabricate the nanotopography, TiO_2_ nanotubes were formed on a pure titanium substrate by using anodic oxidation equipment at different constant voltages (30, 40, 50, 60, and 70 V) for 1 h (Fig. 1). A uniformly distributed array of self-assembled nanotubes was observed by scanning electron microscopy (SEM). Side and top views of the nanotubes are shown in Fig. 2a, b. The height of the nanotubes in all samples in this study was approximately 2 μm, while the inner diameters of the nanotubes were approximately 74 nm (30 V), 92 nm (40 V), 112 nm (50 V), 128 nm (60 V), and 148 nm (70 V) (Fig. 2c). This indicated that the height of the nanotubes was related to the time of anodic oxidation and the inner diameter was related to the voltage of anodic oxidation. X-ray energy dispersive analysis (EDS) was then carried out to analyze the elemental composition of the nanotubes. This showed that the nanotubes consist of only two elements, O and Ti (Fig. 2d, e). Atomic force microscopy (AFM) was used to detect the nanotubular structures and measure the arithmetical mean deviation of the profile of nanotubes as the surface roughness (Ra) (Fig. 2f). The data showed that the surface roughness of nanotubes increased as the diameter (i.e., the voltage of anodic oxidation) increased (Fig. 2g).

### TiO_2_ Nanotubes Induced Osteogenic Differentiation of MSCs

**Fig. 3:**
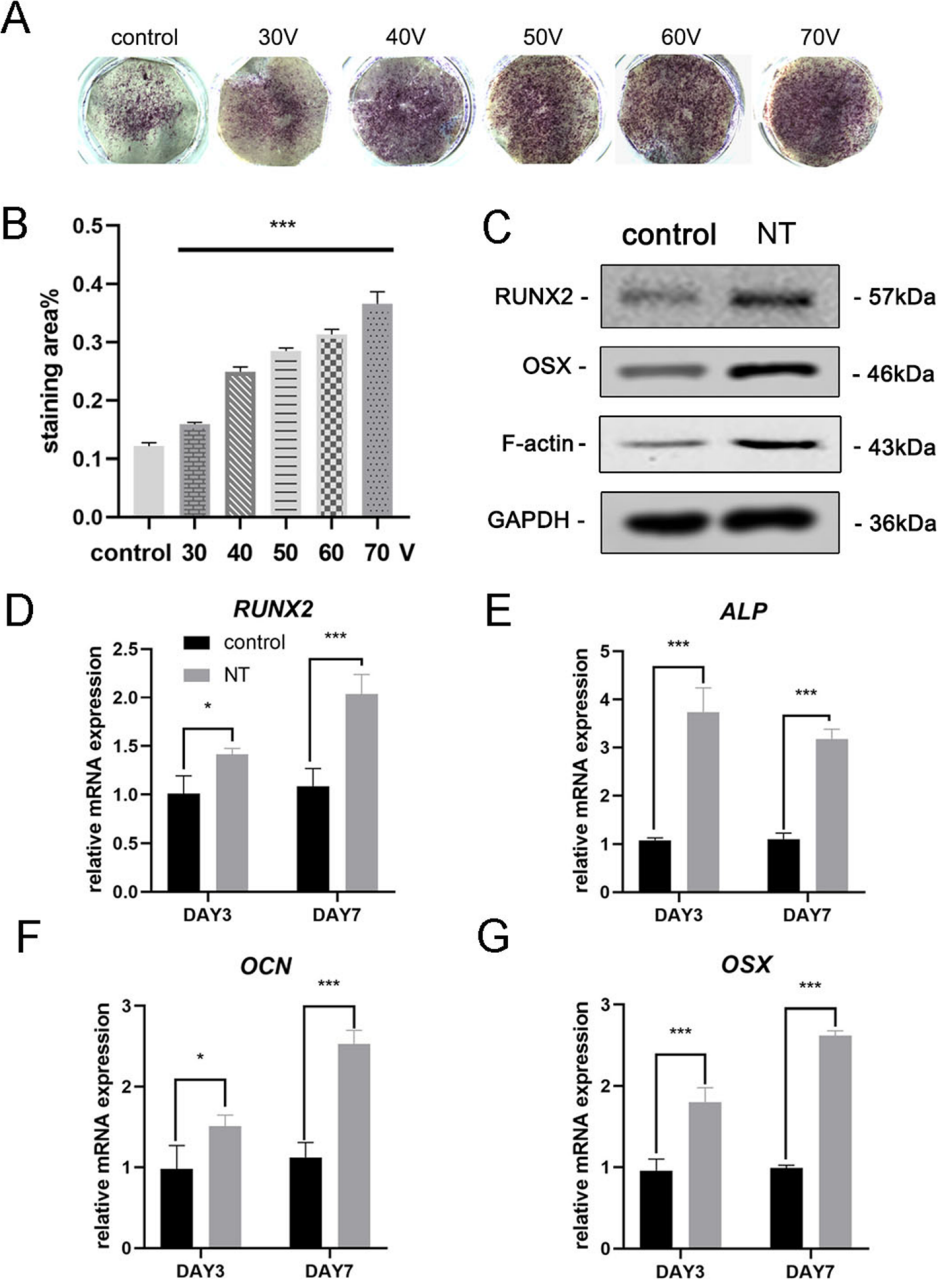
TiO_2_ nanotubes enhanced osteogenic gene expression of BMSCs. **a** ALP staining of the smooth titanium substrate and five different nanotube substrates. The cells were induced with osteogenic medium for 7 days. **b** Statistical analysis of the staining area was performed using ImageJ. **c** Osteogenesis-associated proteins (RUNX2, and OSX) and F-actin in MSCs were analyzed by Western blotting at day 7. The mRNA expression of RUNX2 (**d**), ALP (**e**), OCN (**f**), and OSX (**g**) at days 3 and 7, analyzed by qRT-PCR. *NT* the nanotube group. Data represent the mean ± SD of three samples. *P < 0.05, **P < 0.01, and ***P < 0.001

After 7 days of osteoinduction, ALP staining was first carried out to evaluate osteogenic differentiation of MSCs. The staining results showed that MSCs cultured on TiO_2_ nanotubes had higher ALP activity than cells cultured on a smooth titanium substrate (control group) (Fig. 3a). Statistical analysis of the staining area demonstrated that the ability of nanotubes to induce osteogenic differentiation was significantly enhanced when compared with the control group. Meanwhile, we observed a trend that within the diameter range of this experiment, the larger the diameter of the TiO_2_ nanotubes, the stronger the ability to induce osteogenic differentiation (Fig. 3b). Thus the 70 V group was used in subsequent experiments to better display the results. Next, we analyzed osteogenic gene expression at days 3 and 7. MSCs cultured on TiO_2_ nanotubes for 3 days and 7 days both showed significant promotion of the expression of osteogenic genes (RUNX2, ALP, OCN, and OSX) compared to the control group (Fig. 3d–g). Western blot results confirmed that the protein expression of RUNX2 and OSX also increased after 7 days of osteoinduction (Fig. 3c). Interestingly, we found that F-actin was upregulated in the TiO_2_ nanotubes group. Therefore, it was obvious that TiO_2_ nanotubes directed MSCs toward osteoblast differentiation, which was related to the diameter of the nanotubes. Our results also suggested the involvement of F-actin in this process.

### F-actin Mediated Osteoblast Differentiation of MSCs on TiO_2_ Nanotubes

**Fig. 4:**
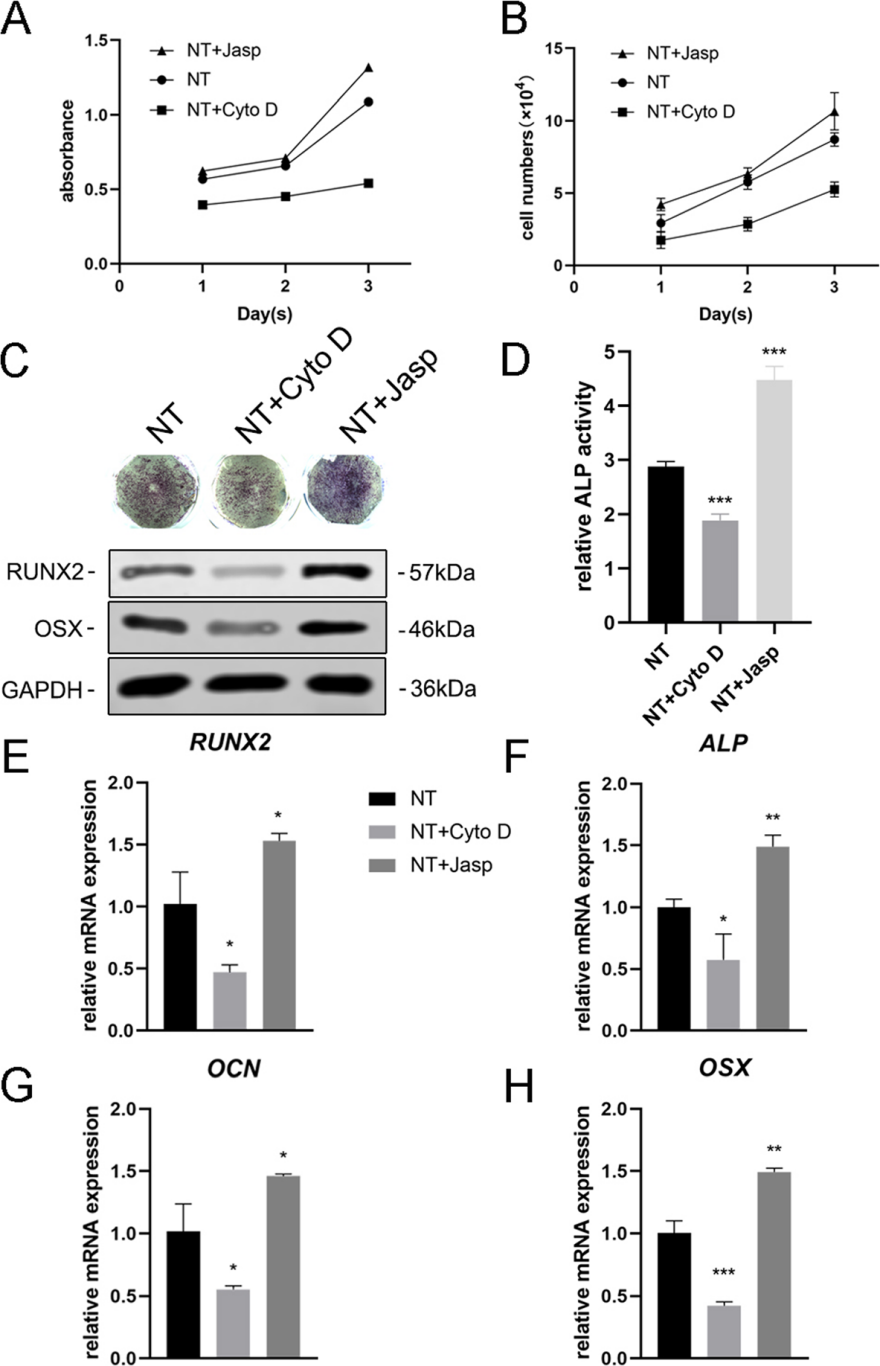
F-actin assembly regulated the expression of osteogenic genes in BMSCs. **a**, **b** Cell proliferation after Cyto D and Jasp treatment was determined using the CCK-8 assay or automated cell counter at days 1, 2, and 3. **c**, **d** ALP staining and ALP activity analysis was performed to evaluate the expression of ALP in MSCs after drug treatment for 3 days. Staining area was analyzed using ImageJ. **c**, **e**–**h** Western blotting and qRT-PCR were used to compare the changes of osteogenesis-related markers in the NT+ Cyto D group and the NT+ Jasp group with those in the control group (without drug treatment). *NT* the nanotubes group. Data represent the mean ± SD of three samples. *P < 0.05, **P < 0.01, and ***P < 0.001

**Fig. 5:**
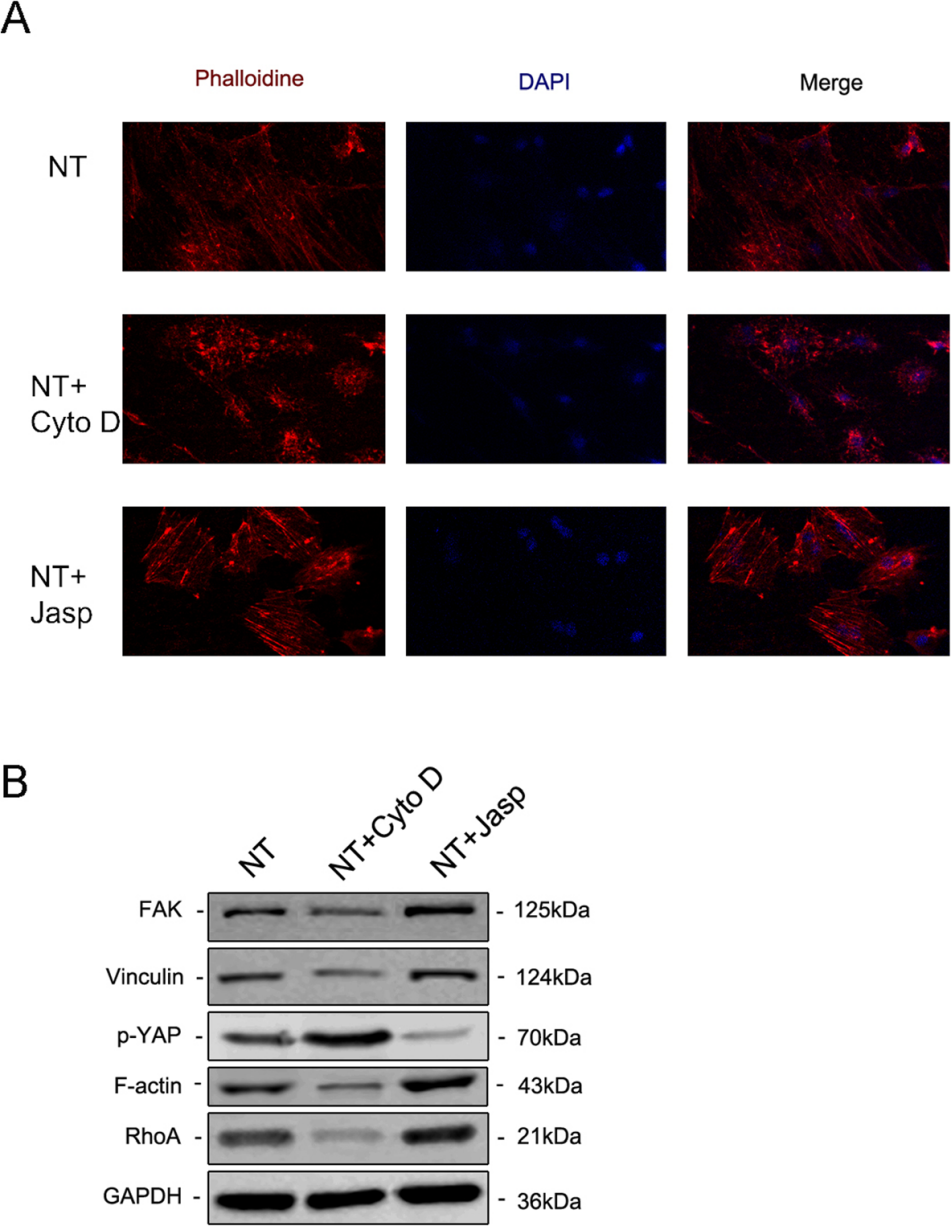
Immunofluorescence staining revealed the level of F-actin by staining with rhodamine-conjugated phalloidin (**a**). The protein expression of FAK and VCL contained in the focal adhesion complex, RhoA and phosphorylated YAP were investigated by Western blotting (**b**). *NT* the nanotubes group

To further explore whether F-actin was involved in nanotopography-induced cell differentiation, we used two reagents, jasplakinolide (Jasp) and cytochalasin D (Cyto D), to regulate F-actin polymerization in a positive and negative way respectively. The confocal photomicrographs of Rhodamine-Phalloidin staining showed that F-actin in the Cyto D treatment group was almost depolymerized and fibrous structures were rarely seen, while Jasp stabilized and polymerized F-actin, verified by more distinct and brighter bundle-like structures than observed in the control group (Fig. 5a). In addition, Western blot analysis also confirmed that the protein expression of F-actin was affected, which proved that Cyto D and Jasp both acted as expected (Fig. 5b). Cell proliferation assay showed that Cyto D significantly inhibited cell proliferation, while Jasp promoted cell growth (Fig. 4a). The cell count results were consistent with this finding (Fig. 4b).

Next, we evaluated the ability of MSCs to differentiate into osteoblasts to investigate whether F-actin mediated this process. We first detected ALP as an early marker of osteogenesis. Compared with the control group, Cyto D treatment reduced the expression of ALP and its activity while that in the Jasp treatment group was upregulated (Fig. 4c, d). Consistent with this result, Jasp treatment resulted in an increase in the protein levels of RUNX2 and OSX while Cyto D had the opposite effect (Fig. 4c). In agreement with this, the mRNA expression levels of osteo-specific genes, including RUNX2, ALP, OCN, and OSX, showed the same trend after drug treatment (Fig. 4e–h). Above all, these data indicated that F-actin played an important role in the process of osteogenic differentiation of MSCs induced by TiO_2_ nanotubes. Promoting F-actin depolymerization inhibited the nanotopography-induced osteoblast differentiation while the stabilization and polymerization of F-actin enhanced osteoblast differentiation.

### F-actin Regulated Osteoblast Differentiation of MSCs on TiO_2_ Nanotubes Through MKL1 and YAP/TAZ

**Fig. 6:**
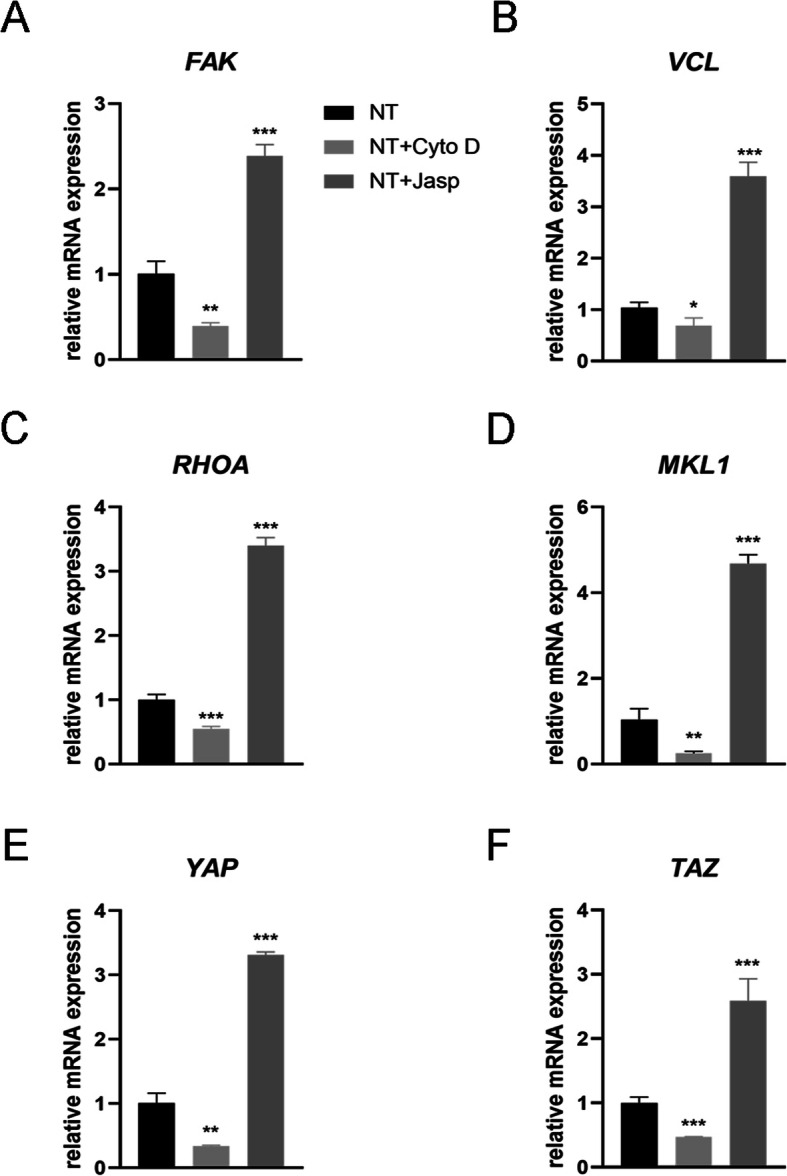
Effect of F-actin assembly on FAK (**a**), vinculin (VCL) (**b**), RhoA (**c**), MKL1 (**d**), YAP (**e**), and TAZ (**f**) gene expression in MSCs. *NT* the nanotubes group. Data represent the mean ± SD of three samples. *P < 0.05, **P < 0.01, and ***P < 0.001

To dissect the underlying mechanism involving F-actin in regulation of the fate of MSCs, we investigated proteins/molecules that directly interact with F-actin or affect F-actin polymerization. First, we tried to identify how the nanotopography affected the balance between F-actin and G-actin. TiO_2_ nanotubes as a physical signal differ from membrane-permeable chemical signals and thus must use some components of the cell membrane to transmit stimuli into the cells. Accumulating evidence indicates that the focal adhesion complex, including integrin, talin, focal adhesion kinase (FAK), vinculin (VCL), tensin, and other proteins, functions as a signal carrier, which informs cells about the condition of the extracellular matrix (ECM) and thus affects their biological behavior [22, 23]. More importantly, F-actin binds to integrins through such focal adhesion complexes, and thereby forms mechanical linkages between intracellular actin bundles and the ECM [24]. Consequently, we next analyzed the expression of components of the focal adhesion complex. Results showed that the protein and mRNA expression of VCL and FAK were consistent with the change of F-actin, indicating that the focal adhesion complex was involved in the process of osteogenic differentiation of MSCs induced by TiO_2_ nanotubes (Figs. 5b and 6a, b). In addition, we also found that RhoA, a small GTPase protein in the Rho family of GTPases, was upregulated in the Jasp treatment group and inhibited by Cyto D (Figs. 5b and 6a, b). RhoA is an important upstream signal transduction molecule in the MAPK pathway, and could be regulated by FAK [25, 26]. The primary function of RhoA is to promote the polymerization and stability of stress fibers (F-actin) and assembly of the focal adhesion complex [27]. Taken together, these data suggested that TiO_2_ nanotubes could influence F-actin polymerization through the focal adhesion complex and RhoA.

But how does F-actin regulate cell fate? Most studies have demonstrated that F-actin is involved in cell migration, cell division, endocytosis, and especially tumor cell invasion [28–30]. Few studies have suggested that F-actin could also regulate cell differentiation, let alone its specific molecular mechanism [31, 32]. Consequently, we searched for articles that mentioned the F-actin changes and found that YAP/TAZ, two closely related transcriptional co-activators in the Hippo signaling pathway, which shuttle between the cytoplasm and the nucleus, may serve as mechanotransducers in regulating MSC differentiation [33–35]. In addition, we also found that MKL1, a key regulator of smooth muscle cell differentiation, which interacts with the transcription factor serum response factor, could bind to G-actin and also circulate between the cytoplasm and the nucleus [21, 36]. Our results ultimately identified the involvement of YAP/TAZ and MKL1 in nanotube-induced osteoblast differentiation mediated by F-actin (Figs. 5b and 6d–f). Interestingly, the protein expression of phosphorylated YAP showed the opposite trend, indicating that not only was the expression of YAP changed, but the phosphorylation of YAP was also changed by Cyto D and Jasp (Fig. 5b). This result was consistent with the report that the phosphorylation of YAP/TAZ could be sequestrated in the cytoplasm [35].

**Fig. 7:**
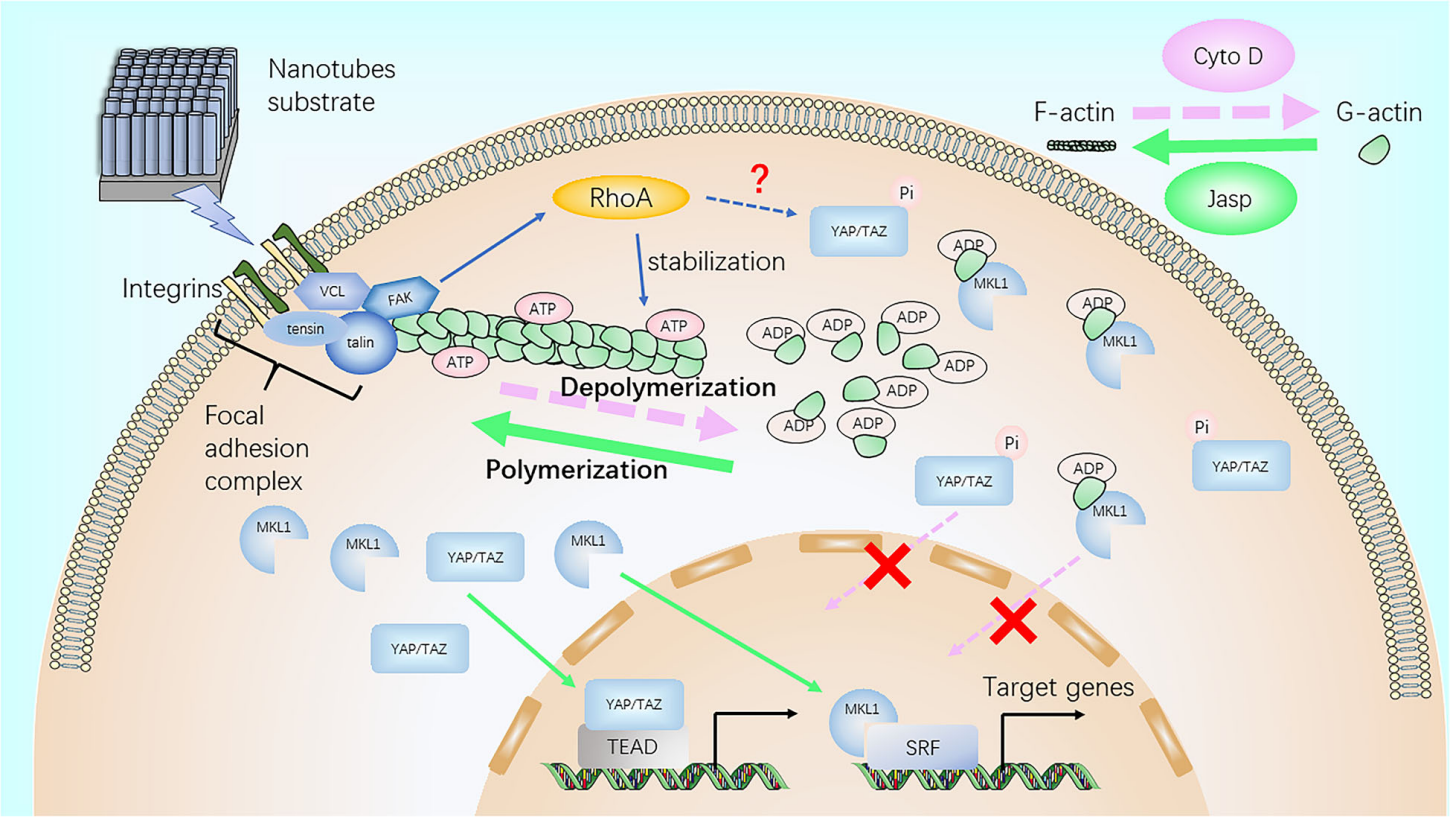
Schematic representation of F-actin assembly induced by nanotubes, and the putative role of MKL1 and YAP/TAZ in acting as the downstream mediators of F-actin signaling to regulate gene expression

In summary, our results preliminarily demonstrated that F-actin regulated osteoblast differentiation of MSCs on TiO_2_ nanotubes through MKL1 and YAP/TAZ (Fig. 7).

## Discussion

Titanium and titanium alloys are the most widely used metal materials in orthopedic clinical implants due to the good properties of titanium [3]. However, aseptic loosening is still an urgent problem to be solved and improved, and the key is likely to lie in improving the integration of the implant and the host bone. Previous studies have shown that surface coating and modification or immobilization of biofunctional molecules will be beneficial to osseointegration [37]. Recently, the surface topography of implants has attracted the attention of many researchers thanks to studies into the cell response to physical cues [9–11, 13, 38]. In this study, we demonstrated the ability of nanotubes to promote osteogenic differentiation of MSCs, and this ability was enhanced with increasing inner diameter of the nanotubes (30–70 V). This will help guide the diameter of the nanotubes on the surface of the implants.

As a topographical structure, nanotubes first change the physical properties of the material, such as adsorption capacity and electrical and thermal conductivity. These physical properties determine their application in the industrial field. For example, most high-voltage power transformers need to be filled with insulating material, which is usually transformer oil or insulating gas. When the insulation of a transformer fails due to overheating and partial discharge, a serious discharge accident will occur. Therefore, finding an effective method that accurately detects the concentration and types of dissolved gases or insulating gas decomposition components in a transformer is necessary to monitor the operating state of the transformer [8, 39–41]. The traditional approach is to look for materials with good gas adsorption in transition elements, which are rich in d electrons, such as Pd(1 1 1) [39]. Nowadays, nanotubes are widely studied for their good gas adsorption properties. He et al. found that CuO-BNNT was suitable for the adsorption of C_2_H_2_, because of its stronger adsorption on C_2_H_2_ [8]. Meanwhile, TiO_2_ itself can be a gas-sensing material. Gui et al. found that Co-doped TiO_2_ further enhanced gas adsorption capacity and exhibited a superior adsorption ability and conductivity change toward C_2_H_4_ molecules [40]. Consistent with this study, Mn-doped graphene also exhibited enhanced conductivity and superior capability of C_2_H_2_ and CO detection than pristine graphene [41]. The above research indicates that the TiO_2_ nanotubes prepared in our experiment have a potential application in the field of monitoring the operative state of a transformer. However, the adsorption capacity and electrical conductivity of the nanotubes to gases need to be further studied, especially whether these properties are enhanced after doping with transition elements (e.g., Mn).

In addition to changing the physical properties of a surface, nanoscale morphology also affects the biological behavior of the cells attached to it. Cells first adhere to the surface of the material and then migrate, proliferate, and differentiate. Compared to a flat surface, the hollow structure of the nanotubes provides fewer adhesion sites for cells. Therefore, in order for the cells to adhere to the nanotube surface steadily and maintain the biomechanical balance within the cell, the focal adhesion complex begins to assemble and mature, and F-actin becomes strong and stable.

F-actin, a linear polymer microfilament consisting of G-actin monomers, is one of the three major components of the cytoskeleton. As a mechanical-loading structure, F-actin is generally believed to be involved in cell division, cell migration, endocytosis, and tumor cell invasion [28–30], but some recent studies showed that it can also affect cell differentiation [31–33, 36]. For example, actin cytoskeletal depolymerization by simvastatin induces chondrocyte differentiation [31], and actin depolymerization enhances adipogenic differentiation in human stromal stem cells [32]. Our results also revealed that, compared with the control group, MSCs cultured on nanotubes had higher F-actin levels and a more obvious fibrous structure. Meanwhile, promotion of F-actin polymerization by Jasp enhanced osteogenic differentiation, while the depolymerization of F-actin inhibited osteogenic differentiation, suggesting that F-actin mediates TiO_2_ nanotube-induced osteoblastic differentiation of MSCs.

F-actin can be regulated by Rho GTPases, members of the Ras superfamily [23, 42], and Rho can induce actin reorganization through at least two effectors, ROCK and Dia. ROCK is activated by binding to Rho-GTP and then myosin light chain (MLC), the substrate of ROCK, plays an important role in F-actin assembly. ROCK inhibits the activity of MLC phosphatase, leading to an increase in MLC phosphorylation, which stimulates the ATPase activity of myosin II and promotes the assembly of F-actin. In addition, ROCK also targets LIM kinase (LIMK). Phosphorylated LIMK inactivates cofilin by phosphorylation, which can disassemble F-actin in its active state. Another effector is Dia, a member of the formin-homology (FH) family of proteins which contains two FH domains. These domains contain multiple proline-rich motifs which bind to the G-actin-binding protein, profilin. This interaction contributes to actin polymerization and F-actin organization [42]. We detected one of the Rho GTPases, RhoA, and found that the expression of RhoA was consistent with the level of F-actin. However, we were unable to clearly describe how the nanotubes regulate the expression of RhoA, because there are many other regulators, including integrin signaling, other adhesion receptors, G protein-coupled receptors (GPCRs), soluble factors such as LPA, receptor tyrosine kinase signaling, and so on [43].

Knowing that F-actin can be regulated by RhoA, we next asked what role focal adhesion played in this process, because focal adhesion complexes, containing integrins, talin, vinculin, paxillin, and focal adhesion kinase (FAK), are formed and mature when cells attach to the surface of nanotubes. Integrins are transmembrane heterodimers that couple the ECM to the other focal adhesion proteins so as to facilitate cell attachment. They not only act simply as hooks but also transmit to the cell critical signals about the nature of its surroundings, which along with other signals such as EGFR, prompt the cell to make decisions about its biological behaviors. These signals are further transmitted to F-actin, which is directly connected to the focal adhesion complexes. On the one hand, the nanoscale morphology causes focal adhesion complex assembly and maturation. On the other hand, kinases such as FAK and Src kinase family members will recruit molecules such as CRK to self-regulate the assembly and maturation of focal adhesion complexes [44–46]. Our results demonstrated that the formation and maturation of focal adhesion complexes were impaired by F-actin depolymerization, suggesting that there was a feedback from focal adhesion complexes to actin assembly in line with published reports.

However, it should not be ignored that these proteins contained in focal adhesion complexes have the function of signal transduction [47]. That is to say, nanotubes may directly regulate gene expression through signal cascades, and F-actin may just participate in or be affected by this process. For instance, the dual kinase complex of FAK and Src can regulate Rho GTPases such as RhoA. This shows that nanotubes can regulate RhoA through integrins and the FAK/Src complex. In addition Src, a non-receptor tyrosine kinase protein, can activate Ras (small GTPase) by phosphorylating FAK at tyrosine residue 925 [47, 48]. Then, Ras activates numerous biochemical pathways, including the well-studied MAPK pathway and the PI3K/AKT/mTOR pathway. In the MAPK pathway, Ras activates c-Raf, followed by mitogen-activated protein kinase kinase (MAP2K) and then MAPK1/2, also known as extracellular signal-regulated kinase (ERK). ERK in turn activates transcription factors such as serum response factor (SRF) and c-Myc that are involved in regulating growth and differentiation [49]. What is more, Runx2, a key transcription factor in osteogenic differentiation, can also be regulated by ERK [50], and our previous study confirmed that mechanical strain promoted osteogenic differentiation of BMSCs through the FAK-Erk1/2-Runx2 pathway [17]. Therefore, we cannot rule out that ERK plays a role in nanotube-induced osteogenic differentiation and further study is still needed.

So what exactly is the role of F-actin in inducing differentiation of nanotubes, because its change can affect cell differentiation? One possibility is that the change of F-actin assembly can inversely regulate the level of FAK so as to induce osteogenic differentiation through the FAK-Erk1/2-Runx2 pathway as described above, because in our results, focal adhesion complexes and actin polymerization showed the same trends of change, indicating that they act as a whole in response to the extracellular environment. However, some other possibilities also exist, and a number of articles have shown that MKL1 and YAP/TAZ act downstream of the actin dynamic balance [20, 51–54]. Both of them shuttle between the cytoplasm and the nucleus, and may help to transduce signals from the cytoskeleton to the nucleus.

MKL1, also termed myocardin-related transcription factor A, is sensitive to changes in G-actin levels. When cytoplasmic G-actin levels increase, monomeric G-actin binds to MKL1 and prevents it from binding to SRF and activating transcription. SRF target genes include actins such as smooth muscle actin (SMA) as well as other actin-binding proteins, including immediate early genes like c-fos and egr1. Recent studies have demonstrated that changing SRF activity could regulate adipogenesis by activating the adipogenesis transcription factor peroxisome proliferator-activated receptor γ (PPARγ), and also regulate bone formation via IGF-1 and Runx2 signaling [55, 56].

YAP and TAZ are two transcriptional coactivators in the Hippo signaling pathway, identified as an important regulatory pathway that restricts cell proliferation, thereby controlling organ size and morphogenesis [20]. Large tumor suppressor genes 1 and 2 (LATS1/2) phosphorylate them, thereby creating a binding site for 14-3-3 proteins, the binding of which prevents their nuclear import [53, 54]. As a consequence, phosphorylated forms of YAP/TAZ are sequestered in the cytoplasm, preventing the expression of genes like Ctgf and Areg. In addition, some studies have shown that YAP/TAZ can interact with T-box 5 (TBX5), RUNX2, and p73 to regulate gene expression [57–59]. Further, cell adhesion to cell matrix proteins has been shown to trigger YAP nuclear localization through an integrin/FAK/Src axis. In our study, the results suggested that this pathway was possibly involved in nanotube-induced differentiation. Further study into the downstream mediators of the integrin/FAK/Src axis should be carried out to clarify the specific mechanism.

On the other hand, more and more studies illustrate that F-actin interacts with Hippo signaling, and somehow inhibits the phosphorylation of YAP [54, 60], which is consistent with our experimental results that promoting F-actin polymerization reduces the expression of phosphorylated YAP. We hypothesize that ATP involved in the process of the transformation between G-actin and F-actin may also play an important role in the phosphorylation of YAP, which is yet to be studied.

After understanding the above possible molecular mechanisms, we can try to explain some of the experimental phenomena found in this study. Our results revealed that the larger the diameter of the nanotubes, the stronger the ability of the nanotubes to promote osteogenic differentiation. This is consistent with previous research [61, 62]. The reason for this phenomenon is that the larger the diameter of the nanotubes, the less adhesion sites they can provide to the cells, and the greater the assembly and maturity of focal adhesion complexes. Along with these, stress fibers made of F-actin will have greater strength and stability. These structures enhance the signaling that promotes osteogenic differentiation. Predictably, however, this effect is significantly reduced when the nanotubes become too large in diameter, making it difficult for the cells to adhere to the surface [12]. Similarly, when the height of the nanotubes is inconsistent, the differences in height can result in a change of adhesion site and rearrangement of the cytoskeleton, which will further affect cell differentiation. Intriguingly, even flat surface materials without nanotube modification can induce changes in cell differentiation. A number of studies have demonstrated that focal adhesion formation and stress fiber organization are regulated by substrate stiffness [63–65], and YAP/TAZ also plays an important role in this process. Therefore, it is obvious that the integrins–FAs (focal adhesions)–F-actin axis plays a role in the transduction of physical signals into intracellular chemical signals.

In summary, our results demonstrated that F-actin regulates osteoblastic differentiation of mesenchymal stem cells on TiO_2_ nanotubes through MKL1 and YAP/TAZ, whose target genes partly explained the proliferation and differentiation of MSCs. We know that there is no single change in the signal network and any change is regulated by numerous molecules and proteins. One type of biological behavior must be the result of the regulation of a series of signaling pathways. Nanotubes induce cell differentiation by triggering a complex network of signals, including integrins, proteins contained in focal adhesion complexes, FAK, Src, Rho GTPase, the MAPK pathway, the Hippo pathway, and other reported signaling pathways. At least as important, there are many signal cycles in the signal network and a downstream signal can regulate the upstream signal via feedback. In this study, we found that vinculin and FAK can be regulated backwards by F-actin assembly, increasing the uncertainty of molecular function. Therefore, more details of the molecular mechanism await further study.

## Conclusions

Our results showed that TiO_2_ nanotubes promoted the osteogenic differentiation of MSCs, and this ability was enhanced with the increasing diameter of nanotubes within a certain range (30–70 V). F-actin mediated nanotube-induced cell differentiation through MKL1 and YAP/TAZ, providing a novel insight into the study of cell differentiation.

## Data Availability

The datasets used and analyzed during the current study are available from the corresponding author on reasonable request.

## Abbreviations

MSCs: Mesenchymal stem cells
SEM: Scanning electron microscopy
EDS: X-ray energy dispersive analysis
AFM: Atomic force microscopy
ALP: Alkaline phosphatase
Cyto D: Cytochalasin D
Jasp: Jasplakinolide
VCL: Vinculin
FAK: Focal adhesion kinase
BCA: Bicinchoninic acid
PVDF: Polyvinylidenedifluoride
Runx2: Runt-related transcription factor 2
Osx: Osterix
OCN: Osteocalcin
YAP: Yes-associated protein
MKL1: Megakaryoblastic leukemia 1
FBS: Fetal bovine serum
ECM: Extracellular matrix
MLC: Myosin light chain
LIMK: LIM kinase
FH: Formin-homology
GPCR: G protein-coupled receptors
MAP2K: Mitogen-activated protein kinase kinase
ERK: Extracellular signal-regulated kinase
SRF: Serum response factor
SMA: Smooth muscle actin
PPARγ: Peroxisome proliferator-activated receptor γ
LATS1/2: Large tumor suppressor gene 1 and 2
TBX5: T-box 5
